# CpG DNA analysis of bacterial STDs

**DOI:** 10.1186/s12879-015-1016-7

**Published:** 2015-07-16

**Authors:** Martin Singer, Dewi J de Waaij, Servaas A Morré, Sander Ouburg

**Affiliations:** Laboratory of Immunogenetics, Department of Medical Microbiology and Infection Control, VU University Medical Center, Amsterdam, The Netherlands; Institute of Public Health Genomics, Department of Genetics and Cell Biology, Research Institutes CAPHRI and GROW, Faculty of Health, Medicine & Life Sciences, University of Maastricht, Maastricht, The Netherlands

## Abstract

**Background:**

Bacterial infections in the genital tract frequently result in morbidity through a variety of inflammation based symptoms. One component of the bacteria that may trigger host inflammatory response is their DNA. CpG motifs in this DNA are known targets for Toll-like receptor 9 (TLR9), which is a pathogen-recognition receptors focusing on CpG DNA. The activation of TLR9 induces the NF-κB inflammatory pathway. This study aims to provide a broad view of the inflammatory potential of CpG DNA motifs in bacteria related to genital diseases: *C. trachomatis*, *E. coli*, *N. gonorrhoeae*, *G. vaginalis*, *H. ducreyi*, *L. crispatus*, *L. gasseri*, *M. hominis*, *M. genitalium*, *T. pallidum*, and *U. urealyticum*.

**Methods:**

Publicly available genomic sequences of the bacterial species and strains have been analyzed *in silico* to produce a CpG index number. This CpG index number shows the relative inflammatory potential of the genome and has previously been used in a study by Lundberg *et al*. (2003). Higher CpG index values suggest a strong CpG induced inflammation potential during infection and vice versa.

**Results:**

The highest observed CpG index belongs to *G. vaginalis* with a value of 26,2, suggesting a strong pro-inflammatory potential when in contact with TLR9. The lowest index belongs to *N. gonorrhoeae* with a value of −79,5, suggesting a strong immunoinhibitory effect on TLR9 contact. Interestingly, *Lactobacilli* showed a mean CpG index value of 4,2, suggesting a weak inflammatory potential.

**Discussion:**

Our results show varying CpG index values between bacterial species. Comparison of CpG indices with the clinical course of several pathogens shows the CpG index helps clarify the clinical course of infection. However, we found no links between CpG index values and either obligate pathogenicity or facultative pathogenicity through bacterial vaginosis. *Lactobacilli* showed relatively low CpG indices which do suggest a lower inflammatory potential from these bacteria.

**Conclusions:**

Our results show varying CpG index values between bacterial species, which may help clarify the clinical course of infection, and may help diagnosis.

## Background

Bacterial Sexually Transmitted Diseases (STD) and Genital Tract Infections (GTI) can cause high levels of morbidity, are often accompanied by social stigma, and are frequently widespread [1]. Symptoms can range from slight inflammation and discharge to infertility and death. In these diseases, inflammatory responses may not always have the positive effect of initiating immune responses to clear the infection [2]. Tissue scarring and an inability to clear bacteria often occur in these infections. Others possess ways of reducing inflammatory response to allow for better survival in the host. The scale of inflammatory response relies on the ability of the host to detect the pathogen and initiate key inflammatory pathways.

One way of initiating inflammation is through the detection of bacterial DNA [3]. Bacterial DNA has unmethylated Cytosine-phosphor-Guanine (CpG) motifs, while mammals generally have methylated CpG motifs which are close to anti-inflammatory DNA sites [4–6]. Unmethylated CpG motifs are targets for the intracellular Toll-like receptor 9 (TLR9) [7]. This receptor is minimally, but consistently expressed in epithelial cells of the genital tract [8, 9]. When TLR9 binds to specific unmethylated CpG motifs it activates the NF-κB pathway, which is a major pathway related to immune response. Activating this pathway initiates a chain reaction resulting in the release of pro-inflammatory cytokines including tumor necrosis factor-α (TNF-α), interleukin-1 (IL-1), IL-6, IL-8, IL-12, and type 1 interferons [4, 10]. These cytokines directly affect the cellular and humoral immune response as well as regulate the inflammation at the site of infection.

As inflammation is a main cause for symptoms in bacterial STDs, we look into how the CpG properties of these pathogens can explain differences in symptoms and outcomes of bacterial STDs, including: *Haemophilus ducreyi*, *Chlamydia trachomatis*, *Neisseria gonorrhoeae*, *Treponema pallidum*, and *Mycoplasma genitalium*.

The first group of pathogens; *C. trachomatis*, *N. gonorrhoeae*, and *M. genitalium* are STDs with similar symptoms and course of infection. These diseases are often asymptomatic, but can also show similar inflammation based symptoms during infection. These can range from mild cervicitis to pelvic inflammatory disease, ectopic pregnancy, and tubapathology associated infertility. It has been shown that activation of the NF-κB pathway through various TLRs is a vital part of the initial immune response to all of these diseases [11–14]. Previous study into the CpG properties of these pathogens showed that *C. trachomatis* serovars C and D have an immunostimulatory effect on the immune system while CpG properties of *N. gonorrhoeae* demonstrates a strong inhibitory potential towards TLR9 binding [12]. *C. trachomatis* serovars E and the highly inflammatory L2b, as well as various strains of *M. genitalium* will be analyzed for the first time in this study.

The second group of pathogens; *T. pallidum* and *H. ducreyi* are STDs that are characterized by ulcers and lesions on the genitals and skin during infection. It has been shown that NF-κB pathway activation through TLR stimulation is vital for initiating an immune response against *T. pallidum* [15, 16]. However, this has not yet been shown for *H. ducreyi* infections. The cellular response to *H. ducreyi*, including macrophages and polymorphonuclear leukocytes, does suggests that cytokine signaling originating from NF-κB pathway activation plays a significant role in the infection [17, 18]. CpG properties indicating a potentially strong activation potential of TLR9 could indicate the primary immune response during infection with these diseases.

Bacterial vaginosis is a disease of the genital tract commonly described as abnormal vaginal discharge, often accompanied with a foul smell, in women of childbearing age. There is no single causative agent of bacterial vaginosis. Instead it is caused by an imbalance in the natural vaginal microflora. One or more commensal bacteria overgrow the naturally dominant Lactobacilli. Some of the bacteria associated with bacterial vaginosis are *Gardnerella vaginalis*, *Mycoplasma hominis*, and *Ureaplasma urealyticum*. An immune response against bacterial vaginosis appears to be lacking. There are no polymorphonuclear leukocytes in the vaginal fluids of women with bacterial vaginosis, however it has been shown that inflammatory cytokines such as IL1 and TNF-α are present [19]. This suggests the imbalance of bacteria is recognized by the immune system, but an effective immune response is inhibited. Host response mechanisms to bacterial vaginosis appear to largely revolve around the activation of the NF-κB pathway [20–22].

Unlike these pathogens, commensal bacteria are naturally found in the host and generally cause no adverse effects. In this study we include the commensal bacteria *Lactobacillus crispatus*, *Lactobacillus gasseri,* and an *Escherichia coli* strain linked to asymptomatic growth in the urinary tract. *L. crispatus* is a beneficial vaginal bacterium whose decrease is characteristic of bacterial vaginosis. The vaginal bacterium *L. gasseri* is also found to protect the vagina from infections. Lactobacilli acidify the vagina and produce hydrogen peroxide which reacts with myeloperoxidase to form reactive molecules toxic to pathogens. Women without vaginal lactobacilli have an increased risk of HIV and gonorrhoeae [23, 24]. It has been shown that *Lactobaccilli* may or may not induce an immune response through the NF-κB pathway on a species dependent basis [25]. *E. coli* is a bacterium generally linked to intestinal inflammation and urinal tract infections. However, *E. coli* can also occur asymptomatically in both the intestines and the urinal tract [26]. The immunopathogenesis of *E. coli* has been clearly linked to the NF-κB pathway, primarily through activation of TLR4 [27, 28]. However this has only been shown for pathogenic strains.

In this study we aim to provide a broader view of the inflammatory properties of bacterial genomes in diseases related to the vaginal or genital tract. These genomes are analyzed *in silico*, to assess the inflammatory potential of CpG motifs in these pathogens, and to predict the role TLR9 plays in the respective host-bacterium interactions and whether strain differences affect this role.

## Methods

Publicly available bacterial genome data has been used for all analyses in this study. NCBI genome databases have been used to obtain the genomes required for analysis. Genomes most focused on by the scientific community that did not have specific uncommon characteristics were chosen for the analysis. Genomes were chosen based on frequency of inclusion in research and lack of traits differentiating them from the usual organism. CpG analysis per genome has been done using previously described genome analysis methods [29]. These methods allowed determination of the amount and build of CpG motifs in a genome, predicted number of CpG motifs when looking at the genomes size, and GC content. The analyzed strains in this study comprise strains of the bacteria: *C. trachomatis*, *E. coli*, *G. vaginalis, H. ducreyi*, *L. crispatus*, *L. gasseri*, *M. genitalium*, *M. hominis*, *N. gonorrhoeae*, *T. pallidum*, and *U. urealyticum* as shown in Table 1.

**Table 1:** Micro-organism names, strains and relevant NCBI references to sequences

Bacteria	Disease	Strain	NCBI reference sequence
*H. ducreyi*	Chancroid	HP35000	NC_017456.1
*C. trachomatis*	Chlamydia	E/11023	NC_017431.1
*C. trachomatis*	Chlamydia	E/150	NC_017439.1
*C. trachomatis*	Chlamydia	E/SW3	NC_017952.1
*C. trachomatis*	LGV	L2b/UCH-1	NC_010280.2
*N. gonorrhoeae*	Gonorrhea	FA 1090	NC_002946.2
*N. gonorrhoeae*	Gonorrhea	NCCP11945	NC_011035.1
*N. gonorrhoeae*	Gonorrhea	TCDC-NG08107	NC_017511.1
*T. pallidum*	Syphilis	DAL-1	NC_016844.1
*T. pallidum*	Syphilis	SS14	NC_010741.1
*T. pallidum*	Syphilis	Chicago	NC_017268.1
*T. pallidum*	Syphilis	Mexico A	NC_018722.1
*M. genitalium*	Non-gonococcal urethritis	G37	NC_017456.1
*M. genitalium*	Non-gonococcal urethritis	M2288	NC_018498.1
*M. genitalium*	Non-gonococcal urethritis	M2321	NC_018495.1
*M. genitalium*	Non-gonococcal urethritis	M6282	NC_018496.1
*M. genitalium*	Non-gonococcal urethritis	M6320	NC_018497.1
*G. vaginalis*	Bacterial vaginosis	409-05	NC_013721.1
*G. vaginalis*	Bacterial vaginosis	ATCC 14019	NC_014644.1
*G. vaginalis*	Bacterial vaginosis	HMP 9231	NC_017456.1
*M. hominis*	Bacterial vaginosis	ATCC 23144	NC_013511.1
*U. urealyticum*	Bacterial vaginosis	ATCC 33699	NC_011374.1
*E. coli*	-	ABU 83972	NC_017631.1
*L. crispatus*	-	ST1	NC_014106.1
*L. gasseri*	-	ATCC 33323	NC_008530.1

### *In silico* analyses

Size and GC content of the analyzed genomes were gathered from the NCBI genome databases. The average amount of CpG hexameres (NNCGNN) per kb of genome was calculated from the total amount of CpG hexameres per genome. CpG hexameres found per genome were compared to the amount of CpG hexameres expected based on the size and the GC content of the genome. We determined the frequency of inflammation stimulatory or inhibitory CpG DNA motifs in their respective genomes [12, 29, 30]. As definition for stimulatory or inhibitory motifs we used published consensus motifs derived from *E. coli* sequences [30]. These comprise inhibitory hexamere motifs NCCGNN and NNCGRN, and stimulatory hexamere motifs RRCGYY. From the difference between these frequencies we produced CpG indices showing the CpG-based immunostimulatory or immunoinhibitory potential of the disease as has previously been described [31, 32].

### Ethics statement

The authors declare that no human material was used during this study.

## Results

Table 2 shows the CpG indices for the examined micro-organisms. An index above zero predicts immunostimulatory properties of the DNA and an index below zero predicts immunoinhibitory properties. The indices do not predict a set amount of inflammation. Larger indices indicate a more potent inflammatory or inhibitory potential. Amount of inflammation belonging to index values can be predicted by comparing scores and *in vitro* or *in vivo* responses.

**Table 2:** Results of *In silico* CpG analyses

Genome					CpG hexamere deviation from expected values in %^a^			
Bacteria	Strain	Size (Mb)	G + C%	CpG per kb^b^	Total CpG^c^	Stimulatory^d^	Inhibitory^e^	CpG index^f^
*H. ducreyi*	HP35000	1.7	38.2	41.0	112.2	124.8	110.4	6.6
*C. trachomatis*	E/11023	1.04	41.3	33.8	79.3	90.7	79.1	3.1
*C. trachomatis*	E/150	1.04	41.3	33.8	79.3	90.8	79.1	3.1
*C. trachomatis*	E/SW3	1.05	41.3	33.8	79.3	90.8	79.1	3.1
*C. trachomatis*	L2b/UCH-1	1.04	41.3	33.9	79.4	86.7	76.1	2.9
*N. gonorrhoeae*	FA 1090	2.15	52.7	92.2	132.9	83.9	143.5	−73.1
*N. gonorrhoeae*	NCCP11945	2.24	52.4	90.7	132.3	80.3	145.8	−78.6
*N. gonorrhoeae*	TCDC-NG08107	2.19	52.5	91.7	132.8	80.5	145.8	−79.5
*T. pallidum*	Dal-1	1.14	52.8	75.1	107.8	107.5	85.7	17.7
*T. pallidum*	SS14	1.14	52.8	75.0	107.7	107.5	85.6	17.7
*T. pallidum*	Chicago	1.14	52.8	75.1	107.8	107.6	85.7	17.7
*T. pallidum*	Mexico	1.14	52.8	75.1	107.8	107.5	85.7	17.7
*M. genitalium*	G37	0.58	31.7	9.7	38.8	74.9	35.0	1.5
*M. genitalium*	M2288	0.58	31.7	9.8	38.9	75.0	35.2	1.5
*M. genitalium*	M2321	0.58	31.7	9.8	39.1	75.3	35.5	1.5
*M. genitalium*	M6282	0.58	31.7	9.8	39.1	74.4	35.5	1.5
*M. genitalium*	M6320	0.58	31.7	9.8	38.9	75.0	35.3	1.5
*G. vaginalis*	409-05	1.62	42.0	48.3	109.3	125.5	87.3	20.2
*G. vaginalis*	ATCC 14019	1.67	41.4	45.0	105.2	138.7	83.3	26.2
*G. vaginalis*	HMP 9231	1.73	41.2	44.4	104.7	137.3	83.1	25.2
*M. hominis*	ATCC 23144	0.67	27.1	12.8	69.4	113.1	70.0	3.8
*U. urealyticum*	ATCC 33699	0.87	25.8	13.8	82.9	138.2	64.3	8.4
*E. coli*	ABU 83972	5.13	50.6	49.2	111.7	146.6	108.2	21.1
*L. crispatus*	ST1	2.04	36.9	27.5	80.8	96.6	80.0	3.7
*L. gasseri*	ATCC 33323	1.89	35.3	23.4	75.4	99.0	73.0	4.6

^a^Deviations in amounts of CpG hexameres compared to the expected amount based on GC content

^b^CpG hexameres occurring per 1 kb of DNA

^c^Total number of CpG hexameres compared to the expected amount

^d^Number of stimulatory CpG hexameres (RRCGYY) compared to expected amount

^e^Number of inhibitory CpG hexameres (NCCGNN and NNCGRN) compared to the expected amount

^f^Index calculated from the difference between stimulatory deviation and inhibitory deviation indices, multiplied by the total CpG index, normalized by multiplying with the amount of CpG hexameres per 1 kb

*G. vaginalis* has the highest index with one strain reaching a value of 26.2, and a mean value of 23.9. Both the included *E. coli* strain and *T. pallidum* also appear to have larger than average mean CpG values, with mean values of 21.1 and 17.7, respectively. The lowest index belongs to *N. gonorrhoeae* with one strain having a CpG value of −79.5 and a mean CpG value of −77.1. *N. gonorrhoeae* was the only bacteria showing a negative CpG value in the analysis.

**Fig 1:**
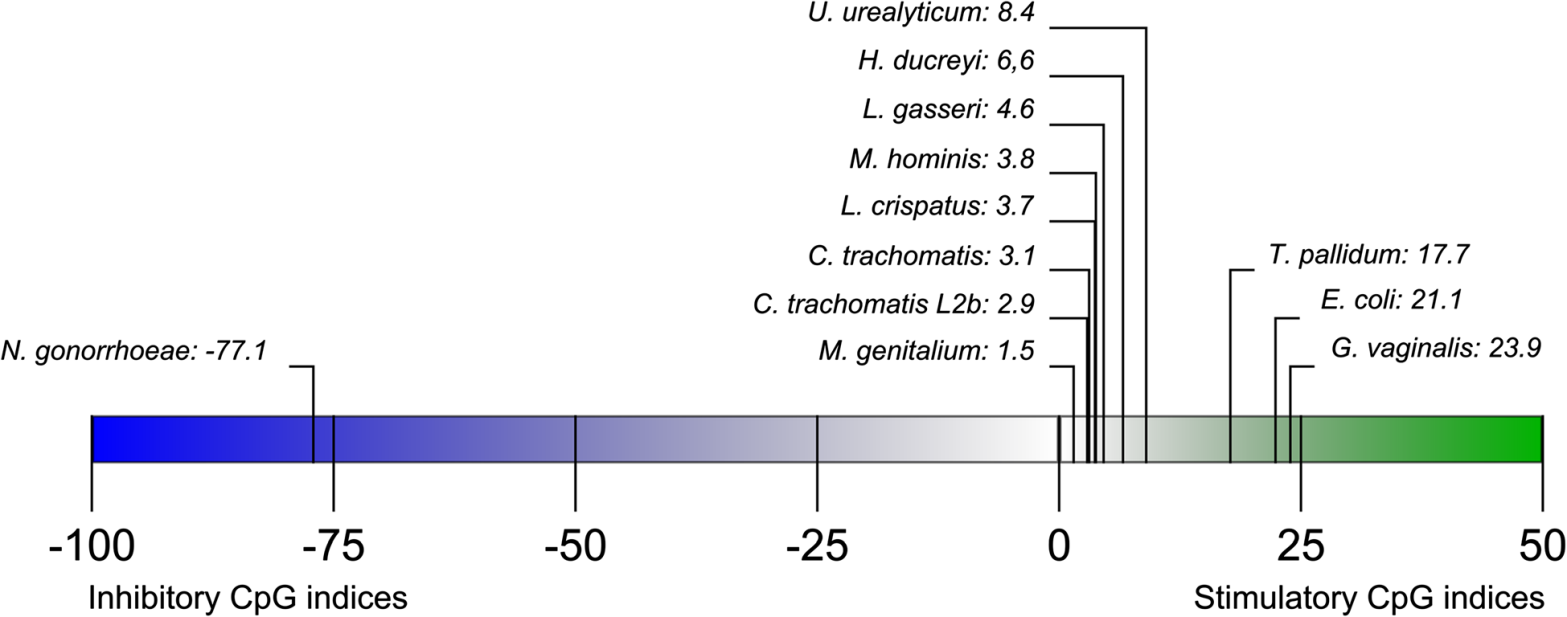
Scale bar showing the position of the mean CpG values per pathogen

A large cluster of genomes were found to have relatively low mean CpG values of <10. The mean CpG value of *C. trachomatis* strains that were not L2b was 3.1, with the included L2b strain showing a slightly lower CpG value of 2.9. *H. ducreyi* showed a CpG value of 6.6. The two included *Mycoplasma* species, *genitalium* and *hominis*, were found to have mean CpG values of 1.5 and 3.8, respectively. The single strain of *U. urealyticum* was found to have a CpG value of 8.4. Lastly, the Lactobacilli were found to have index values of 3.7 and 4.6. Figure 1 shows the mean CpG index values for every pathogen on a CpG axis.

## Discussion

The immune response to bacterial STDs is primarily initiated through activation of TLRs. TLR9 is likely to be a big factor due to activation of the receptor by bacterial DNA CpG motifs. This study has produced an overview of potential TLR9 activation through inflammation stimulating or inhibiting CpG motifs related to a variety of bacterial STDs, bacteria linked to bacterial vaginosis, and commensal bacteria found in the genital tract.

The group of bacterial STDs with symptoms largely related to symptoms induced by inflammation in the host was a likely target to show high potential TLR9 activation through the CpG indices. Surprisingly, *C. trachomatis*, *N. gonorrhoeae*, and *M.genitalium* do not show any indices higher than 3.1. Although there is a relatively low CpG index for both *C. trachomatis* serovars E and L2b, cervical and colonic epithelial cells infected with *C. trachomatis* do secrete pro-inflammatory cytokines in relatively large amounts [33]. Additionally, IL-1α secreted by endocervical epithelial cells was previously found to amplify the inflammatory response by stimulating additional cytokine production without activating more TLRs [34]. A study by Ouburg *et al.* shows that TLR9 does not influence the susceptibility to acute *Chlamydia* infection [12]. This information plus the relatively low stimulatory CpG index of 3.1 of *C. trachomatis* may indicate that it elicits an immune response via another route than TLR9. TLR4 is a likely alternative candidate, as it recognizes chlamydial LPS via its coreceptor CD14 [35]. Similarly, the highly inflammatory strain L2b/UHC-1 shows a comparable CpG index also suggesting that TLR9 and CpG DNA are not vital factors in inflammation during *C. trachomatis* infection*.*

Similarly to *C. trachomatis*, symptoms during infection with *N. gonorrhoeae* are also largely based on inflammation. However it was found to have an inhibitory CpG index of −73.1, similar to findings in a study by Ouburg *et al*. [12]. It has been described that *N. gonorrhoeae* uses several strategies to avoid the immune system. The CpG index of −73.1 of *N. gonorrhoeae* may explain that this pathogen suppresses Th1 and Th2 responses by reducing binding with TLR9 and activation of NF-κB [36]. This reduces the secretion of IL-4 and IL-12 that are needed to activate the Th1 and Th2 responses. Reports show that shortly after infection, CD4+ T cell and CD8+ T cell levels declined [37]. This may also explain why *N. gonorrhoeae* demonstrates an asymptomatic clinical course in most cases [38]. Based on these studies, it is likely that when inflammatory symptoms arise during *N. gonorrhoeae* infection, it is likely through activation of the immune response without activation of TLR9.

*M. genitalium* was found to have a minimal CpG index of only 1.5. Comparing this with the minor CpG index for *M. hominis* of 3.8 and a higher CpG index of 8.4 for the closely related *U. urealyticum* some similarities can be seen. The values suggest minor inflammatory properties of the micro-organisms’ DNA and significance of TLR9 in the inflammatory response to these organisms. No research has been done on the roles of either CpG or the TLR9 pathway in the bacteria. However, a previous study did indicate TLR1 and TLR2 to induce the NF-κB pathway in *M. genitalium* [14]. Therefore, we suggest that activation of the immune response is largely initiated through these pathways instead.

The bacterial STDs *H. ducreyi* and *T. pallidum*, both characterized by the formation of lesions or ulcers as symptoms, showed CpG indices of 6.6 and 17.7, respectively. During *H. ducreyi* infections, increased secretion of TLR9 related pro-inflammatory cytokines including IL-12 and IFNγ would activate and increase differentiation of Th1 cells. A Th1 cellular immune response is needed for clearance of *H. ducreyi* [39]. The effect of some point mutations in *TLR9* on activation of the cellular immune response was shown by Sanders *et al.*, showing a protective effect of *TLR9* + 2848 in a study targeting bacterial meningitis [31]. Unpublished data from our group showed a protective association for *TLR9* + 2848*G and a significant risk enhancing effect for *TLR9* -1237*T plus *TLR9* + 2848*A during *H. ducreyi* infections (manuscript in preparation). This indicates TLR9 activation through CpG motifs in *H. ducreyi* DNA is vital for a proper immune response to this infection

Similarly during *T. pallidum* infections a cellular immune response is vital for clearance of the infection [40]. With pro-inflammatory cytokines being found inside lesions, indicating activation of the NF-κB pathway plays an important role in the initial immune response as well as activation of the cellular response. The relatively high CpG index of 17.7 found for *T. pallidum* indicates that TLR9 can be the primary inducer of the NF-κB pathway during infection with *T. pallidum*.

*G. vaginalis* was found to have the highest mean CpG index of 25.7, suggesting that it has DNA with significant inflammatory properties. However, bacterial vaginosis because of *G. vaginalis* overgrowth only produces mild inflammatory signs. *G. vaginalis* has previously been found to create a biofilm and from there induce controlled inflammation, using the host’s immune response to further its infection [41]. Additionally, Ghione *et al.* has found that a Th2 response activating B-cells produces antibodies specific to *G. vaginalis* influencing the infection but not clearing it [42]. We suggest that the high CpG index found in this study can be explained as part of the way *G. vaginalis* gains advantage from the inflammation while inside a biofilm.

The commensal bacteria *L. gasseri* and *L. crispatus* show a stimulatory effect on the immune system. In contrast to our findings, a study by Ghadimi *et al.* describes that the binding of the commensal bacteria *L. rhamnosus* to TLR9 elicits an intracellular signaling cascade in a manner that reduces the expression of IL-8. TNF-α is being attenuated by reducing IkBα and p38 phosphorylation, which are downstream signaling proteins in the NF-κB pathway [43]. Additionally, recent findings suggest that there is a species specific effect on the inflammatory response of the host to *Lactobaccillus spp*. [25]*.* For example *L. iners* was found to induce pathogen recognition receptor activity and expression of pro-inflammatory cytokines. Conversely, *L. crispatus* was found to not exhibit these effects. This suggests a potential disparity between different *Lactobaccillus* species that may explain the different findings. Indeed, one study found that cytokine production differed between *Lactobacillus* species, and that this cytokine response is primarily due to activation of TLR9 [44]. This may indicate that the relatively small difference between the two species examined here is a fluctuation that apparently has an *in vivo* effect on the production of TLR9 related cytokines.

In contrast to the relatively low CpG indices of the examined *Lactobacilli*, the examined *E. coli* strain showed a high immunostimulatory CpG index of 21.1. Although studies into commensal *E. coli* strains have primarily focused on TLR4 and TLR5, one study has shown cytokine expression profiles during stimulation of TLR9 with commensal *E. coli* DNA linked to NF-κB activation [45–47]. It is strange then, that the presence of the *E. coli* strain does not lead to symptoms that normally occur during *E. coli* pathogenic infections. Previous analysis of the *E. coli* ABU 83972 genome found that the innate immune response of the host is modified during infection with this bacterium [26]. Specifically the IL-1 and IL-6 signaling pathways are affected. The authors suggest that the bacteria uses this modified immune response to adapt on a host-specific basis, to a point where both host and bacterium can benefit from the commensal growth. Therefore, in this specific strain of *E. coli* the immunostimulatory potential of the high CpG index is successfully circumvented.

Comparing our results to previous studies into CpG indices of microbial organisms allows us to put the CpG indices into context [31, 32]. Lundberg *et al.* examined viral DNA to find CpG indices up to 148.7 for Bovine Herpesvirus-1 and a low of −9.4 for Epstein Barr virus. They suggest that viral DNA characteristics make it hard to compare CpG indices of these viruses, and mention that the results may have been affected by the CpG motifs used for analysis, as they were determined from bacterial DNA. Nevertheless, they showed a predictive value in the CpG index as the negative results relate to low inflammation in clinical infections and relatively high results relate to strong inflammatory responses *in vivo* [32]. The study of Sanders *et al*. focused on bacterial meningitis and can be better related to this study. Interestingly, their analyses of *N. mengitidis* resulted in a CpG index of −106.8, suggesting a very strong immunoinhibitory relation similar to the one found in this study for *N. gonorrhoeae. H. ducreyi* has a CpG index of 6.6, only 0.6 points removed from *H. influenzea* with an index of 7.2. Sanders *et al*. relate even the weak CpG indices to clinical inflammation during their respective diseases [31].

Looking at the clinical pictures of pathogens included in this study, the bacteria *H. ducreyi* and *T. pallidum* cause visible soars or ulcers during their clinical course while *C. trachomatis, N. gonorrhoeae,* and *M. genitalium* have the shared characteristic of causing tubal pathology, which in all cases can lead to infertility and ectopic pregnancy. The ulcer and lesion producing group has CpG indices that are overall higher than the group of pathogens related to tubapathology, even though the clinical course of the last group of diseases shows clear inflammation in the host. However, previous studies have shown that pathogens related to tubapathology are detected more accurately through other pathways. The difference in CpG index values in this group may be explained by the fact that *C. trachomatis* is intracellular, and *N. gonorrhoeae* extracellular, thus the two are exposed to different immunological factors. This has already been shown for *M. genitalium,* which is detected through TLR1 and TLR2 instead [14]. There are also two non-pathogenic groups of bacteria studied here. The first is the commensal group including *L. crispatus*, *L. gasseri*, and an asymptomatic *E. coli* strain. The second is the bacterial vaginosis group consisting of *G. vaginalis*, *M. hominis*, and *U. urealyticum*. These may show symptoms like increased vaginal discharge, change of smell, and itchiness [48]. These two groups both show widely varying positive CpG indices depending on the examined organism. This indicates that TLR9 initiation potential is likely highly specific to an organism, and related to multiple factors such as interaction with the immune system. Additionally, it suggests that CpG/TLR9 interaction alone cannot account for all specific inflammatory symptoms. A previous study has shown that bacterial CpG specifically induces the proinflammatory cytokines IL-6, IL-12, and Interferon γ [49]. However, the symptoms created during infection with the included organisms are formed by a complex system including both host and bacterial factors for which the CpG index value reflects the intensity of the initial inflammation.

Including all the studied bacteria into one biological model is difficult, as many of these bacteria have different ways of avoiding or interacting with the immune system. However, the comparison of CpG indices with clinical outcomes of the diseases showed that there are similar characteristics between some bacteria. As was previously mentioned, positive CpG indices result in stimulation of TLR9, which activates the TLR9 related NF-κB pathway. At the end of this pathway, upregulated transcription of NF-κB targeted genes causes more inflammatory cytokines such as IL-1 and TNF-α to be released. We suggest that a relatively low or negative CpG index still allows the DNA of the bacteria to bind. However, this DNA then does not stimulate TLR9, or does not stimulate TLR9 as strongly into activating the NF-κB pathway. Conversely, a positive CpG index means the DNA binds to TLR9 more easily or activates the NF-κB pathway in a stronger manner.

Reflecting back on this study some strengths become clear. The methods used in this study have previously been shown to have significant predictive value. This study is also the first to look at CpG DNA and its effect on inflammation for such a large group of relevant bacteria in the genital tract. However, some limitations do apply. Though the predictions have previously been shown to have significant value, *in vitro* study is needed for verification. Also this study has only looked at sequenced strains. Therefore some results may not be in line with what can be seen in infections with current wild type strains in *in vivo* infections. Additionally, the used CpG sequences were all derived from studies on *E. coli*. There is no study into whether these sequences act like stimulatory and inhibitory motifs for all the bacteria studied here or if there are any additional relevant sequences.

This study has indicated inflammatory potential in bacterial STDs through analysis of the bacterial genomes. If this result can be corroborated *in vitro* it can clarify the immunopathogenesis for the bacteria studied here. In the future this data can be used to specifically focus research into inflammation during infections with the studied bacteria. Additionally, results found in this study can be used to compare indices of other micro-organisms studied using the same methods.

## Conclusion

In conclusion our results show varying CpG index values between bacterial species. Comparison of CpG indices with the clinical course of several pathogens shows the CpG index helps clarify the clinical course of infection. However, we found no links between CpG index values and either obligate pathogenicity or facultative pathogenicity through bacterial vaginosis. *Lactobacilli* showed relatively low CpG indices which do suggest a lower inflammatory potential from these bacteria.

## Abbreviations

CpG: Cytosine-phosphor-Guanine group
GTI: Genital tract infection
IL: Interleukin
STD: Sexually transmitted disease
TLR: Toll-like receptor
TNF: Tumor Necrosis Factor
